# Early life stress induces decreased expression of CB1R and FAAH and epigenetic changes in the medial prefrontal cortex of male rats

**DOI:** 10.3389/fncel.2024.1474992

**Published:** 2024-10-22

**Authors:** Arijana Demaili, Anna Portugalov, Mouna Maroun, Irit Akirav, Katharina Braun, Jörg Bock

**Affiliations:** ^1^Department of Zoology/Developmental Neurobiology, Institute of Biology, Otto von Guericke University Magdeburg, Magdeburg, Germany; ^2^Department of Psychology, School of Psychological Sciences, University of Haifa, Haifa, Israel; ^3^The Integrated Brain and Behavior Research Center (IBBR), University of Haifa, Haifa, Israel; ^4^Sagol Department of Neurobiology, Faculty of Natural Sciences, University of Haifa, Haifa, Israel; ^5^Center for Behavioral Brain Sciences (CBBS), Magdeburg, Germany; ^6^PG Epigenetics and Structural Plasticity, Institute of Biology, Otto von Guericke University Magdeburg, Magdeburg, Germany

**Keywords:** early life stress, pefrontal cortex, endocannabinoid system, endocannabinoid receptor 1, epigenetics

## Abstract

Several studies in both animal models and in humans have provided substantial evidence that early life stress (ELS) induces long-term changes in behavior and brain function, making it a significant risk factor in the aetiology of various mental disorders, including anxiety and depression. In this study, we tested the hypothesis that ELS in male rats (i) leads to increased anxiety and depressive-like symptoms; and (ii) that these behavioral changes are associated with functional alterations in the endocannabinoid system of the medial prefrontal cortex (mPFC). We further assessed whether the predicted changes in the gene expression of two key components of the endocannabinoid system, cannabinoid receptor 1 (CB1R) and the fatty acid amide hydrolase (FAAH), are regulated by epigenetic mechanisms. Behavioral profiling revealed that the proportion of behaviorally affected animals was increased in ELS exposed male rats compared to control animals, specifically showing symptoms of anhedonia and impaired social behavior. On the molecular level we observed a decrease in CB1R and FAAH mRNA expression in the mPFC of adult ELS exposed animals. These gene expression changes were accompanied by reduced global histone 3 acetylation in the mPFC, while no significant changes in DNA methylation and no significant changes of histone-acetylation at the promoter regions of the analyzed genes were detected. Taken together, our data provide evidence that ELS induces a long-term reduction of CB1R and FAAH expression in the mPFC of adult male rats, which may partially contribute to the ELS-induced changes in adult socio-emotional behavior.

## Introduction

The aetiology of the most prevalent psychiatric disorders, including pathological anxiety and major depressive disorder, can be linked to a disturbed development and functional maturation of neuronal circuits during the perinatal period (McEwen, 2006; Hovens et al., 2012; Menke et al., 2018). Early critical phases of brain development are characterized by an increased sensitivity to adverse environmental influences, including early life stress (ELS; Lupien et al., 2009; Bock et al., 2014). ELS has been shown to affect synapse formation and the maturation of neural circuits and neurotransmitter systems (Bock et al., 2014; Derks et al., 2016; Guadagno et al., 2021), which is assumed to result in dysfunctional stress responses and disturbed socio-emotional behavior later in life (Heim et al., 2008; Gerritsen et al., 2017). It has been suggested that individuals, which are experiencing early adverse life events such as childhood trauma, abuse and neglect show an increased risk for social anxiety at later life (Kuo et al., 2011). Also, there is evidence that adverse events in childhood are implicated in the development of major depression (Kuzminskaite et al., 2022; Negele et al., 2015), anxiety (Kascakova et al., 2020; Huh et al., 2014) and attention-deficit/hyperactivity disorder (Brown et al., 2017; Peleikis et al., 2022) later in life.

The endocannabinoid system (ECS) has emerged as an important factor in regulating neural and behavioral responses towards adverse stimuli and as a modulator of synaptic plasticity (Viveros et al., 2007; Dow-Edwards and Silva, 2017; Winters and Vaughan, 2021). Furthermore, experiments involving pharmacological interventions targeting ECS activity have revealed its therapeutic potential for treating psychological disorders, including depression and anxiety (Fowler, 2015; Bedse et al., 2018; Schmidt et al., 2021; Paulus et al., 2021). Endocannabinoids (eCBs), such as N-arachidonoylethanolamine (anandamide, AEA; Devane et al., 1992) and 2-arachidonoylglycerol (2-AG; Mechoulam et al., 1995; Sugiura et al., 1995), are a group of endogenous lipid mediators and neurotransmitters that are released “on demand.” eCB signalling is controlled in part by selective eCB-degrading enzymes, such as fatty acid amide hydrolase (FAAH), which is responsible for the degradation of AEA and monoacylglycerol lipase (MAGL), involved in the degradation of 2-AG (Egertová et al., 1998; Romero et al., 2002; Maccarrone, 2017). The synaptic effects of endocannabinoids are mediated by endocannabinoid receptors 1 (CB1R) and 2 (CB2R). CB1R is located on presynaptic excitatory and inhibitory terminals and is involved in retrograde synaptic signalling (Castillo et al., 2012). CB1R is found in abundance in limbic and cortical regions, such as the hippocampus, amygdala and prefrontal cortex, where its activity, mediated by degrading enzymes like FAAH, is involved in the orchestration of emotional and cognitive behavior (Katona et al., 2006; Mechoulam and Parker, 2013; Hu and Mackie, 2015).

So far, the long-term consequences of ELS exposure on endocannabinoid function are not fully understood. Also, the detailed mechanisms underlying the long-term effect of ELS exposure on behavior and brain function remain still unclear. Epigenetic modifications are believed to be key mediators between early adverse life experiences and enduring changes in gene expression (Bock, 2024; Demaili et al., 2023; Kocamaz et al., 2022; Köhler et al., 2019; Bock et al., 2014; Li et al., 2020; Alyamani and Murgatroyd, 2018; Vaiserman, 2015).

Cortical–limbic circuits are particularly vulnerable towards stress exposure, likely due to their relatively late and gradual neuronal and synaptic development. In a series of experiments in different animal models we demonstrated that the mPFC, including the anterior cingulate cortex, prelimbic cortex and infralimbic cortex is particularly sensitive to ELS and prenatal stress exposure (Portugalov et al., 2022; de Schultz et al., 2020; Braun et al., 2020; Bock et al., 2017; Bock et al., 2016; Bock et al., 2014; Bock et al., 2005). In our recent study in the mPFC of female rats we showed that ELS-induced changes of CB1R and FAAH gene expression are mediated by long-term changes in promoter-specific DNA-metylation and are permanent and still detectable in adulthood (Demaili et al., 2023).

To expand these findings, the overarching aim of the present study was to investigate the long-term consequences of ELS exposure on adult socio-emotional behaviors and on endocannabinoid function in adult male rats. Specifically, we tested the hypothesis that: (i) ELS induces long-term changes in the gene expression of two main regulatory components of the eCB system, CB1R and FAAH, in the mPFC; and (ii) these long-term changes in gene expression are mediated by alterations in DNA methylation and/or histone acetylation.

## Materials and methods

### Animals

Sprague–Dawley (SD) rat dams and pups (Envigo, Israel) and pups were housed in the animal facility of the School of Psychological Sciences at the University of Haifa. The rats were maintained on a 12:12 light/dark cycle with food and water provided *ab libitum*, in a polypropylene cages (59 × 28 × 20 cm), at a room temperature 22 ± 2°C. The day of birth was defined as postnatal day 0 (PND0). Pups were weaned on PND21 and arbitrarily assigned to same sex groups (4–5 animals per cage). This study focused on male rats, while female rats were used for the parallel experiments (Demaili et al., 2023). All experiments were approved by the University of Haifa Ethics and Animal Care Committee and appropriate measures were taken to minimize pain and discomfort (approval numbers: 741/2020, 765/2021).

### Early life stress

For ELS, we used the established paradigm of maternal neglect (Avishai-Eliner et al., 2001) with modifications (Alteba et al., 2016, 2020, 2021; Portugalov et al., 2022). Briefly, from PND7 to PND14, dams and their pups were housed in a cage with limited bedding material, specifically a 1.2 cm layer of “Sunny Chips.” Before PND7 and after PND14 until weaning at PND21, dams and their litters were reared under standard conditions.

### Experimental groups

Male rats were arbitrarily assigned to one of two experimental groups:

Control (CON): The animals were not exposed to ELS and were group-housed together with their mother in a cage with adequate bedding material (7–9 cm layer) until weaning.

Early life stress (ELS): Animals in this group were exposed to the ELS paradigm as described above from PND7 to PND14.

### Behavioral analysis

**Figure 1:**
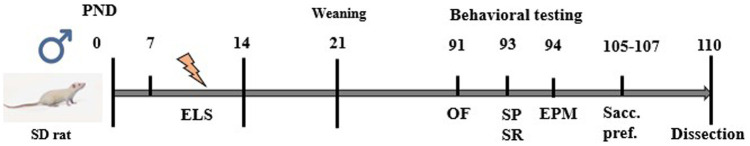
Experimental design. Male SD rats were subjected to ELS during PND 7–14. In adulthood, rats were subjected to a behavioral test battery, starting with Open field test (OF) at PND 91. At PND 93, Social preference (SP) and Social recognition (SR) tests were performed. Elevated plus maze test (EPM) was performed a day later, at PND 94. Last behavioral test was Saccharine preference test (Sacc. pref.), performed for 3 days between PND 105 and 107. At PND 110 rats were sacrificed and mPFC was dissected.

Rats were exposed to a battery of behavioral tests in adulthood (PND 90–105) (Figure 1). Testing was performed under dim lighting (15–20 lx) between 13:00 and 16:00 h to reduce stress and facilitate more accurate observations of the animals’ behavior. We have consistently applied this lighting condition across behavioral experiments in previous studies (Portugalov et al., 2022; Bright and Akirav, 2023). Rats were arbitrarily assigned to the experimental groups (CON, *n* = 16; ELS, *n* = 14).

#### Behavioral profiling

In order to categorize the stress-exposed animals as “affected” or “unaffected,” behavioral profiling was conducted, as previously described (Ardi et al., 2016; Albrecht et al., 2021; Sarkar et al., 2022; Demaili et al., 2023). Parameters from the behavioral tests included in the analysis were: Open field test (time spent in the center, total distance and freezing time), Elevated plus maze (time spent in the open arms, time spent in the closed arms, total distance and freezing time), Saccharine preference test (saccharine consumption), Social preference (time spent with social peer, discrimination index), Social recognition (time spent with familiar peer, discrimination index). The performance of the control group was considered representative of an “unaffected normal population” and average values and standard deviations were calculated to establish the upper and lower cut-off values for each parameter. Stress-exposed animals were classified as “affected” if their performance deviated beyond these cut-off values in four parameters or more.

#### Locomotor activity and anxiety-like behavior in the open field test

Open field test was the first test of the behavioral battery, done at PND 91 (Figure 1). This test was conducted in a square black open field (50 × 50 × 50 cm). The floor was divided by 1 cm wide white lines into 25 squares measuring 10 × 10 cm each. The open field arena was thoroughly cleaned between each trial. The movements of the rats were recorded and analysed for 15 min using a video tracking system (Ethovision × T 14.0, Noldus Information Technology) to measure locomotor activity, quantified as the distance moved in cm. Time spent in the center area (s) during the first 5 min was used as an indicator of anxiety-like behavior (Bauminger et al., 2022).

#### Social preference and social recognition

Social preference and social recognitions tests were done both on PND 93 (Figure 1). This task aimed to assess sociability and short-term social memory. The “partner” rat was confined to a separate section of the open field (50 × 50 × 50 cm) and placed in transparent perforated Plexiglas panel (corrals; Portugalov et al., 2022). During a 5 min preference phase, the rat was allowed to explore a novel juvenile rat and a novel object. For the 5 min recognition phase, after 30 min in a holding cage, the rat was allowed to explore the familiar juvenile and a novel juvenile rat, both confined to the corrals. The trials were videotaped (Dericam, Indoor Pan/tilt IP camera M801W, USA). Discrimination indices were calculated to assess (a) social preference: time exploring the novel juvenile/total exploration time (object + juvenile rat) × 100% and (b) social recognition: time exploring the novel juvenile/total exploration time (familiar + novel juvenile) × 100%.

#### Activity and anxiety-like behavior in the elevated plus maze (EPM)

Elevated plus maze test was conducted on PND 94 (Figure 1). The test apparatus consisted of a black plus-shaped maze (110 × 110 cm, 70 cm above the floor), with two opposing open arms and two opposing closed arms. The rat was placed in the center of the maze and allowed to explore the maze freely for 5 min. The movements of the rats were recorded and analysed using a video tracking system (Ethovision × T 14.0, Noldus Information Technology) to measure locomotion and exploratory activity, quantified as the distance moved in cm. To assess anxiety-like behavior the time spent in the closed arms (in seconds) and the total distance traveled (cm) were measured.

#### Saccharine preference test

Saccharine preference test was done during a period of 3 days, from PND 105 to PND 107 (Figure 1). This test aimed to assess depressive-type behavior (anhedonia). Before the dark phase of the cycle, water bottles were removed and replaced with two bottles, one containing water and the other containing a 0.3 mg/L solution of saccharine. Saccharine consumption was measured during the dark cycle (12 h) and then normalized to each rat’s body weight. The saccharine preference ratio was calculated as the saccharine consumption/total consumption (saccharine consumption + water consumption) × 100%. Measurements of saccharine preference were taken three times, with a 24-h interval between each measurement, and the average of all the measurement was calculated for each rat.

### Tissue preparation

Animals from the respective experimental groups were sacrificed 3 days after the last behavioral test, on PND110. Brains were rapidly frozen in liquid nitrogen and stored at −80°C. For gene expression analysis, tissue from right and left medial prefrontal cortex (mPFC) tissue was dissected. The samples were obtained by punches from frontal sections at the level of the prefrontal cortex, corresponding to the following anatomical coordinates: Bregma + 2.70; Interaural + 11.70 (Paxinos and Watson, 2006). Collected samples included tissue from the anterior cingulate cortex (Acd, Cg1), prelimbic cortex (PrL) and infralimbic cortex (IL). Total RNA was extracted using the innuPREP RNA Mini Kit 2.0 (Analytik Jena, Berlin, Germany) following the manufacturer s instructions. Genomic DNA contaminants were removed using the innuPREP DNase I Digest Kit (Analytik Jena, Berlin, Germany). For analysis of gene expression, DNA-methylation, histone 3 global acetylation and histone 3 lysine 27 acetylation animals were arbitrarily assigned to the respective experimental groups (CON, *n* = 9; ELS, *n* = 10). Also for analysis of promoter specific histone 3 acetylation (nChIP) animals were arbitrarily assigned to the two experimental groups (CON, *n* = 6; ELS, *n* = 6).

### Gene expression analysis

One-step quantitative real-time PCR was performed using the QuantiNova Multiplex RT-PCR kit (Qiagen GmbH, Hilden, Germany) and Taqman gene expression assays (Life Technologies, Carlsbad, CA, USA) as described previously (Demaili et al., 2023). 20 ng of RNA was used as a starting material in a reaction volume of 20 μl. Commercially available rat Taqman probes were used for endocannabinoid receptor 1 (eCB1R, *cnr1* gene) and fatty acid amide hydrolase (FAAH) as target genes (Rn00562880_m1 for *cnr1* and Rn00577086_m1 for *faah*) and hypoxantine guanine phosphoribosyl transferase (HPRT) as reference gene (Rn01527840_m1). The qPCR reaction was carried out with real-time PCR cycler Rotor-Gene Q (Qiagen GmbH, Hilden, Germany). Cycling conditions were as follows: Denaturation, 95°C for 2 s.; Combined annealing/elongation 60°C for 30 s., total 40 cycles. Relative gene expression was calculated using the delta–delta Ct method (Livak and Schmittgen, 2001). All samples were run in two independent experimental replicates, with each gene assay performed in triplicates. CB1R and FAAH gene expression is presented as fold change relative to the respective mean value of the control group.

### DNA-methylation analysis

Deoxyribonucleic acid (DNA) methylation levels were determined using the bisulfite-converted DNA and pyrosequencing method, as described previously (Demaili et al., 2023; Kocamaz et al., 2022). In brief, genomic DNA was extracted from left mPFC using DNeasy Blood and Tissue Kit (Qiagen GmbH, Hilden, Germany) following the manufacturer’s instructions. EpiTect Bisulfite Kit (Qiagen GmbH, Hilden, Germany) and QIAcube (Qiagen GmbH, Hilden, Germany) were used for DNA bisulfite conversion and cleanup, respectively. Analyzed CpG sites within circumscribed DNA sequences in the promoter regions of the CB1R and FAAH genes were identified by using the National Center for Biotechnology Information (NCBI) databank. Primers covering 11 CpG sites in the promoter region of the CB1R gene were individually designed using PyroMark Assay Design Software (Qiagen GmbH, Hilden, Germany). For the FAAH gene, 2 primers covering 9 CpG sites within the promoter region were used, which are commercially available as PyroMark CpG assays (Qiagen). Pyrosequencing was performed with Pyromark Q96 ID (Qiagen GmbH, Hilden, Germany). For verification of methylation results, CpGenome rat methylated and unmethylated genomic DNA standards (Merck KGaA, Darmstadt, Germany) were used. DNA methylation levels were automatically analyzed using PyroQ CpG software (Qiagen GmbH, Hilden, Germany). The results are presented in two ways: (i) mean value of methylation levels across 11 analyzed CpG sites for CB1R and across 9 analyzed CpG sites for FAAH and (ii) CpG site specific methylation levels at the analyzed individual CpG sites.

### Analysis of global histone modifications

Global histone 3 acetylation (H3ac) and histone 3 lysine 27 acetylation (H3K27ac) levels were determined by quantitative western blot (WB) analysis, as described previously (Köhler et al., 2019). In short, tissue samples were homogenized in extraction (lysis) buffer (0.1 M Tris/HCL pH 8.0; 0.01 M EDTA; 10% SDS); 1× Halt Protease Inhibitor Cocktail (Thermo Fisher Scientific, Waltham, MA, USA). After cell lysis, we continued with sonification (ultrasonic vibration), to isolate nuclei. Protein concentration was measured using the Bio-Rad DC™ Universal Protein Assay Kit II (Bio-Rad, Hercules, CA, USA), and was in a range between 10 and 20 μg/μL. Gel electrophoresis was performed using 30 μg of protein with loading buffer and Bio-Rad Tris-Glycine Mini-PROTEAN TGX Precast Gels (Bio-Rad, Hercules, CA, USA). Size marker PeqGOLD Protein Marker V (PeqLab/VWR International GmbH, Darmstadt, Germany) was used to determine the protein molecular weight. After gel electrophoresis, the samples were blotted onto a nitrocellulose membrane (GE Healthcare, Chalfont St Giles, UK). The blots were blocked with RotiBlock BSA blocking reagent (Carl Roth, Karlstuhe, Germany), followed by overnight incubation with primary antibodies: anti-acetyl H3 (#06-599, 1:10,000; Merck Millipore, Billerica, MA, USA), anti-acetyl H3K27 (#39133, 1:2,000; Active Motif, Carlsbad, CA, USA) and antihypoxanthine phosphoribosyltransferase I (HPRT1, #PA-522281, 1:500; Thermo Fisher Scientific, Waltham, MA, USA) at 4°C. The antibodies specific for histone 3 have been used in our previous study (Köhler et al., 2019). The next day, blots were incubated for 1-h at room temperature with a secondary antibody (horseradish peroxidase conjugated anti-rabbit; #12–348; 1:4,000; Merck Millipore, Billerica, MA, USA). Horseradish peroxidase was detected with Luminata Crescendo Western HRP substrate (Merck Millipore, Billerica, MA, USA). Syngene G:Box system and Gene Tools software (Syngene Europe, Cambridge, UK) were used for signal detection and data analysis, respectively. H3ac/HPRT and H3K27/HPRT ratio is presented as fold change relative to the mean value of the respective control group.

### Analysis of promoter specific histone 3 acetylation

Histone 3 acetylation on CB1R and FAAH gene promoters was quantified by native chromatin immunoprecipitation (nChIP). NChIP protocol was performed following an established protocol (Xie et al., 2013; Köhler et al., 2019) with specific modifications for rat brain tissue (Donovan and Lichota, 2014). In short, nuclei were extracted using a glass homogenizer and a combination of extraction buffers: buffer 1; 0.4 M sucrose, 10 mM Tris–HCl pH = 7.9, 5 mM sodium butyrate, protease inhibitor cocktail (Thermo Fisher Scientific, Waltham, MA, USA), buffer 2; 0.25 M sucrose, 10 mM Tris–HCl pH = 7.9, 5 mM sodium butyrate, protease inhibitor cocktail (Thermo Fisher Scientific, Waltham, MA, USA), 1% Triton X-100, 10 mM MgCl_2_. Digestion step was done with digestion buffer; 0.32 M sucrose, 50 mM Tris–HCl pH = 7.5, 5 mM sodium butyrate, protease inhibitor cocktail (Thermo Fisher Scientific, Waltham, MA, USA), 4 mM MgCl_2_, 1 mM CaCl_2_ and with 30 U MNase (Thermo Fisher Scientific, Waltham, MA, USA). The enzymatic digestion was stopped by adding 0.5 M EDTA. Sample was centrifuged at 11,600 *g*, 4°C for 5 min. Supernatant was divided and mixed with anti-acetyl-H3 antibody (#06–599, Merck Millipore, Billerica, MA, USA), then incubated overnight at 4°C with constant rotation. For immunoprecipitation protein A/G Magnetic Beads were used (Thermo Fisher Scientific, Waltham, MA, USA). The beads and antibody-sample mixture were incubated at 4°C for 3 h while rotating. Beads were then collected on magnet and washed with following buffers for 10 min each: Low salt wash buffer (150 mM NaCl, 0.1% SDS, 1% Triton X-100, 2 mM EDTA, 20 mM Tris–HCl pH 7.9), high salt wash buffer (500 mM NaCl, 0.1% SDS, 1% Triton X-100, 2 mM EDTA, 20 mM Tris–HCl pH 7.9) and 3× TE buffer (10 mM Tris–HCl, pH 7.5, 1 mM EDTA). After washing steps, samples were eluted in freshy prepared elution buffer (1% SDS, 0.1 M NaHCO_3_), for 2 × 15 min. After the last elution step, antibody-bound samples and inputs were incubated with 0.5 M EDTA, 1 M Tris–HCl pH 6.8 and 20 μg Proteinase K (Thermo Fisher Scientific, Waltham, MA, USA) for 3 h at 45°C in a thermomixer. After proteinase K treatment, DNA was extracted with phenol/chloroform/isoamylalcohol (Carl Roth GmbH, Karlsruhe, Germany) and subjected to DNA purification, by MinElute Reaction CleanUp Kit (Qiagen GmbH, Hilden, Germany). The results were analysed by ChIP quantitative real-time PCR, using the QuantiNova Multiplex RT-PCR kit (Qiagen GmbH, Hilden, Germany) and custom-made TaqMan assays targeting CB1R (FAM-coupled) and FAAH (VIC-coupled; Thermo Fisher Scientific, Waltham, MA, USA). For both CON and ELS groups, 6 animals were used per group. All the samples were run in three independent experiments and in duplicates within one experiment. Fold enrichment in histone H3 acetylation on the CB1R and FAAH gene promoter regions was calculated with formula: 2^{−[Ct(H3ac) − Ct (input)]}.

### Statistics

Differences between the experimental groups for individual behavioral tests, gene expression, mean DNA methylation, CpG site specific methylation, H3ac and H3K27ac were analysed using a two-tailed, unpaired *t*-test analysis. Behavioral profiling was conducted as described above and the distribution of affected versus unaffected populations was calculated by using Pearson’s chi-squared test. All statistical calculations were performed using GraphPad Prism 8 (San Diego, CA, USA), with the exception of behavioral profiling, which was analyzed using SPSS 27 (IBM, Chicago, IL, USA). All values were presented as mean value ± SD. *p* < 0.05 was considered statistically significant. No sample size calculation was performed. Number of subjects for behavioral analysis as well as for molecular changes was estimated based on previous studies from our groups (Demaili et al., 2023; Portugalov et al., 2022; Kocamaz et al., 2022). All animals for the respective methodological approaches were used for statistical analysis and thus no outlier test were performed.

## Results

### Behavioral analysis

#### Behavioral profiling

**Figure 2:**
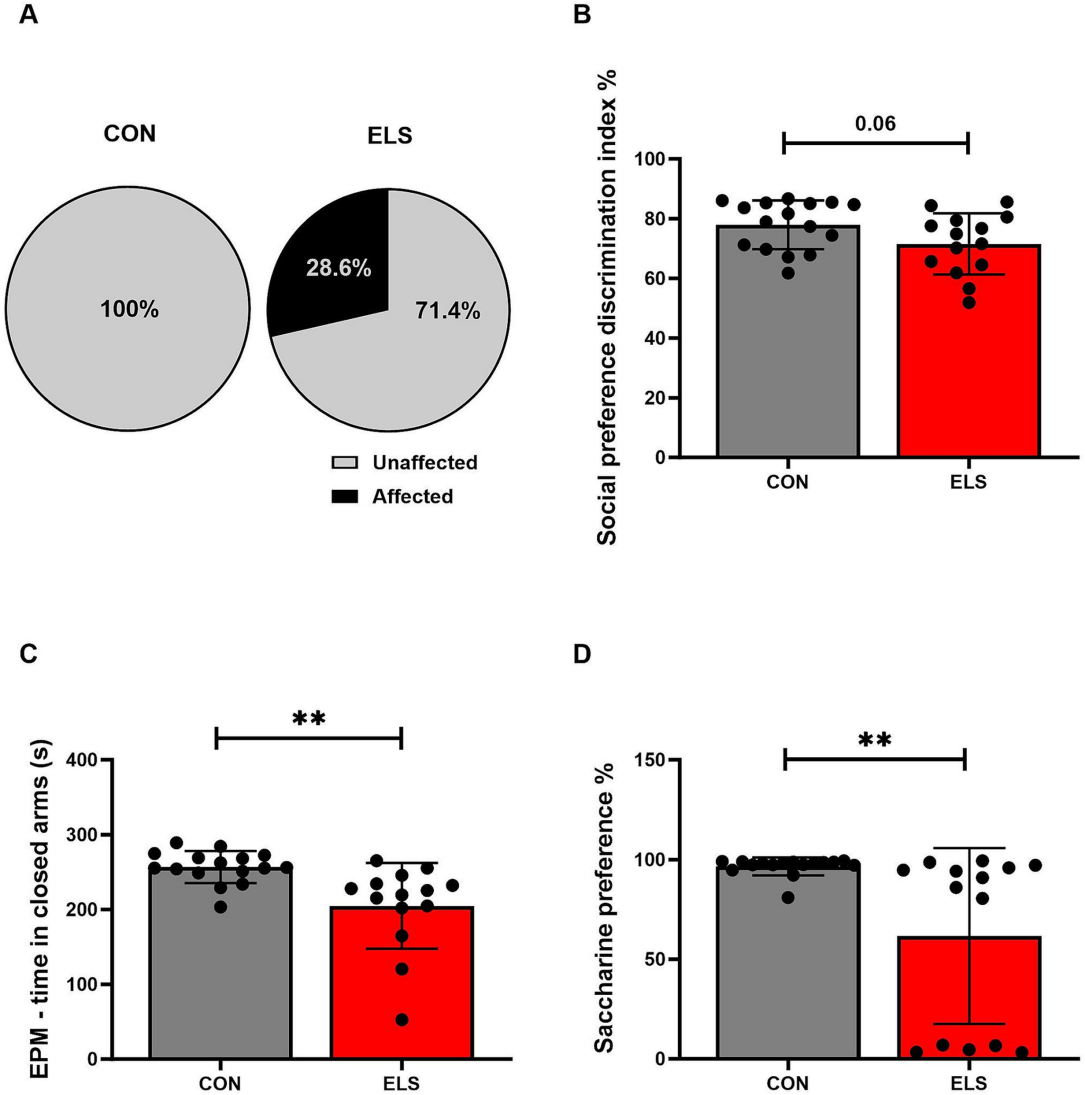
Long-term consequences of ELS exposure on adult socio-emotional behavioral traits. The effects of ELS on different behavioral parameters were calculated using Pearson’s chi-squared test for behavioral profiling **(A)** and two-tailed, unpaired *t*-tests for individual behavioral parameters **(B–D)**. Significant results are indicated as *p ≤ 0.05 and **p ≤ 0.01. **(A)** Results of behavioral profiling, with affected and unaffected animals presented as percentage distributions. **(B)** Social preference discrimination index. **(C)** Time spent in closed arms in the EPM. **(D)** Saccharine preference. Social preference discrimination index was calculated as time exploring the novel juvenile/total exploration time (object + juvenile rat) × 100%. The saccharine preference ratio was calculated as the saccharine consumption/total consumption (saccharine consumption + water consumption) × 100%. All bars depict mean ± SD. CON, control (*n* = 16); ELS, early life stress (*n* = 14).

Behavioral profiling of individual animals revealed that the behavioral phenotype of adult males was affected by ELS exposure. The analysis included all parameters of the behavioral test battery described above. Chi-Square test revealed that in the ELS group there were significantly more affected animals when compared to controls (Chi^2^ = 5.275, *p* = 0.0216; Figure 2A).

To further explore the contribution of specific behavioral traits in more detail, all behavioral tests were statistically evaluated individually.

#### Social preference test and social recognition test

For the social preference test discrimination index, two tailed, unpaired *t*-test analysis revealed a non-significant but strong trend of ELS males to show lower preference for a novel social partner when compared to CON group [t_(28)_ = 1.906, *p* = 0.0669; Figure 2B]. For the social recognition test discrimination index no significant effect was detected [t_(28)_ = 0.375, *p* = 0.7105].

#### Elevated plus maze and open field test

For the EPM, an unpaired *t*-test analysis revealed that ELS males spent significantly less time in the open arms than CON males [t_(28)_ = 3.38, *p* = 0.0022; Figure 2C]. For time spent in the open arms of EPM, a non-significant trend towards more time spent in open arms for ELS males, compared to CON was found [t_(28)_ = 1.77, *p* = 0.0877].

For the parameter of locomotor activity in the EPM, no significant effect was detected [t_(28)_ = 0.275, *p* = 0.7849].

In the OF, no significant effects for locomotor activity [t_(28)_ = 0.492, *p* = 0.6263] neither for time in the center [t_(28)_ = 0.126, *p* = 0.9007] were found.

#### Saccharine preference test

For saccharine preference test, a two-tailed, unpaired *t*-test revealed that ELS males showed a significantly lower saccharine preference, compared to CON [t_(28)_ = 3.159, *p* = 0.0038; Figure 2D].

### Gene expression

#### ELS exposure decreased the expressions of cannabinoid receptor 1 mRNA and fatty acid amide hydrolase mRNA in the mPFC

**Figure 3:**
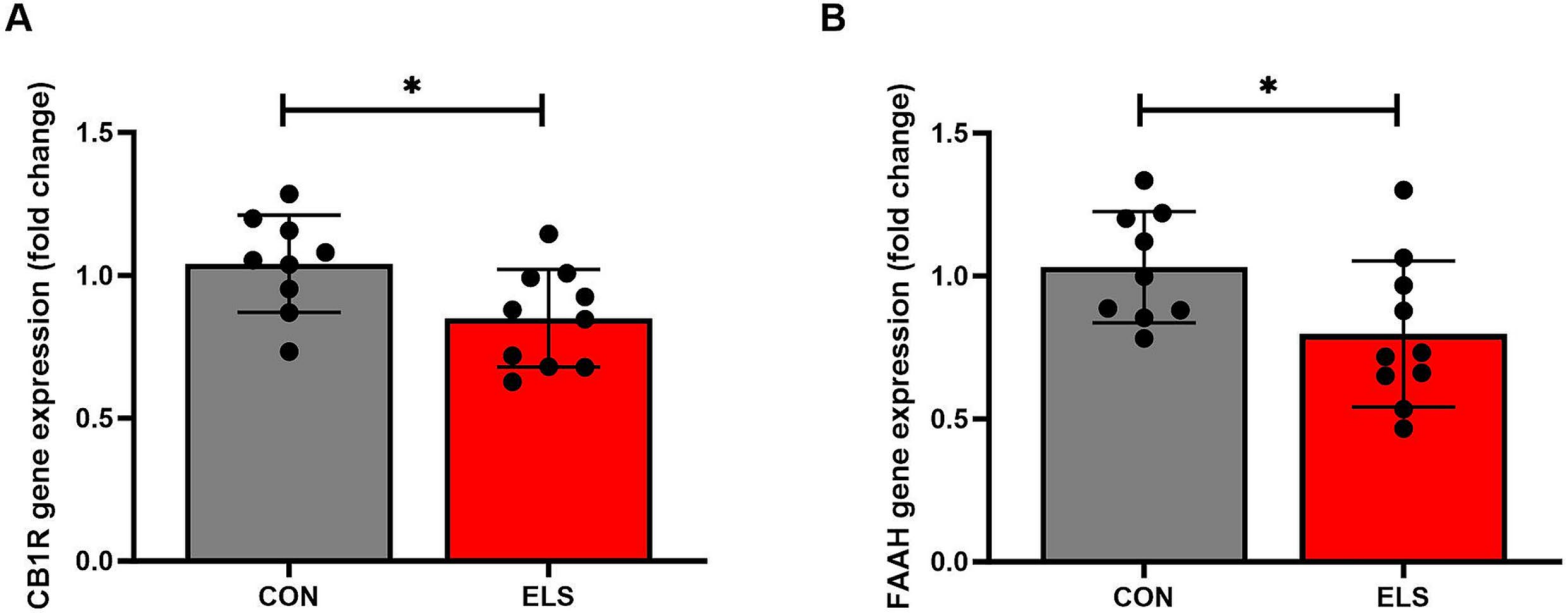
ELS decreased cannabinoid receptor 1 (CB1R) and fatty acid amide hydrolase (FAAH) relative gene expression in the medial prefrontal cortex (mPFC) of adult male rats. Gene expression data are calculated as 2^–DDCt^ values and presented as relative values to the mean value of the control group ± SD. CB1R results are shown in **(A**); FAAH results are shown in **(B)**. Significant results of two tailed, *t*-test are presented as *p ≤ 0.05. CON, control (*n* = 9); ELS, early life stress (*n* = 10).

For CB1R gene expression, a two tailed, unpaired *t*-test analysis showed a significant decreased expression levels in the ELS group, compared to the CON group [t_(17)_ = 2.431, *p* = 0.0264; Figure 3A].

Similarly, for relative FAAH gene expression, a significantly decreased expression levels were detected in the ELS group, compared to the controls [t_(17)_ = 2.221, *p* = 0.0402; Figure 3B].

### DNA methylation

#### CB1R and FAAH DNA methylation levels were unaltered after ELS

##### CB1R

**Figure 4:**
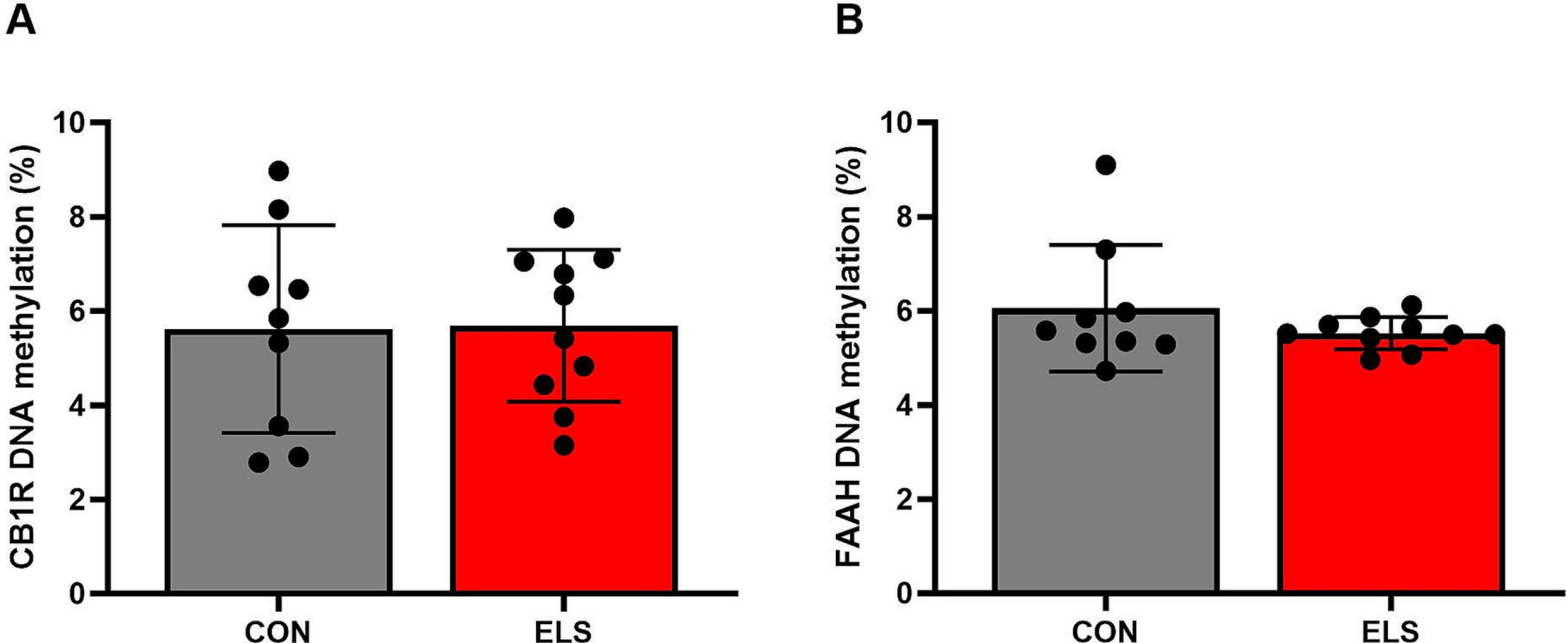
Mean DNA methylation levels across the analyzed 11 CpG sites within the promoter region of the CB1R gene **(A)** and across the analyzed 9 CpG sites within the promoter region of the FAAH gene **(B)**. Mean methylation was calculated as mean value of DNA-methylation across 11 CpG sites of CB1R gene and across 9 CpG sites of FAAH gene. DNA methylation levels are presented as mean ± SD. CON, control (*n* = 9); ELS, early life stress (*n* = 10).

**Figure 5:**
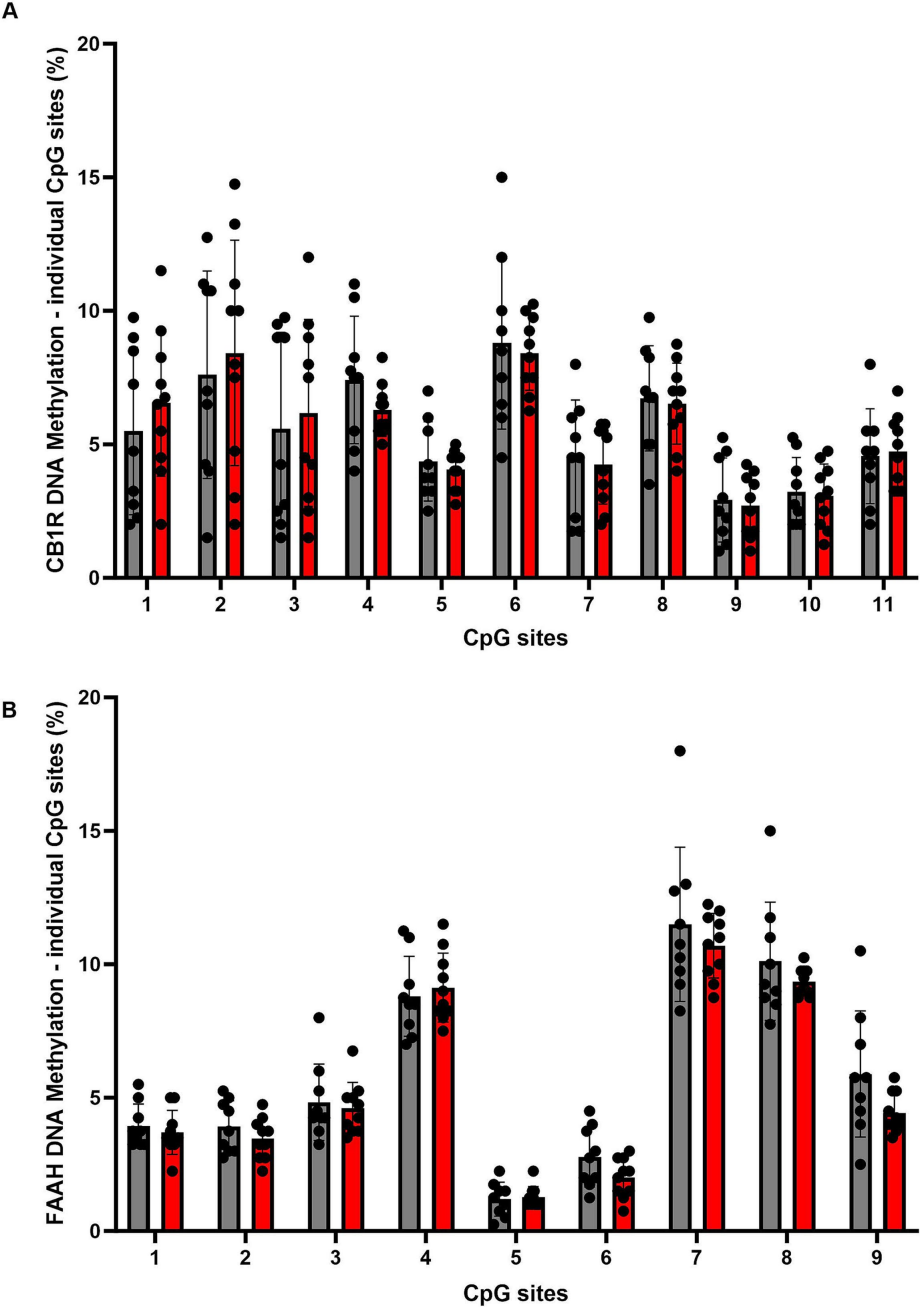
Overview of DNA methylation levels at individual CpG sites within the promoter region of the CB1R gene **(A)** and the FAAH gene **(B)**. Data represent CpG-site specific DNA-methylation: for CB1R gene, 11 individual CpG-sites, for FAAH gene 9 individual CpG-sites were analyzed. CpG-sites are shown as ordinal numbers. DNA methylation levels are presented as mean ± SD. CON, control (*n* = 9); ELS, early life stress (*n* = 10).

For DNA methylation analysis, 11 CpG sites within the promoter region of the CB1R gene were measured, and mean methylation levels were analyzed. A two tailed, unpaired *t*-test of mean methylation levels across 11 CpG sites revealed no statistically significant difference between the CON and ELS groups [t_(17)_ = 0.0779, *p* = 0.9388; Figure 4A]. Also, for each of the analyzed 11 CpG sites individually revealed no statistically significant differences between the CON and ELS groups were detected (Figure 5A; see Table 1 for details).

**Table 1:** Unpaired *t*-test analysis of 11 individual CpG sites within the promoter region of the CB1R gene.

Individual CpG sites	*t*-test results (non-significant = ns)
1	ns [t_(17)_ = 0.7795, *p* = 0.4464]
2	ns [t_(17)_ = 0.4357, *p* = 0.6686]
3	ns [t_(17)_ = 0.3622, *p* = 0.7217]
4	ns [t_(17)_ = 1.392, *p* = 0.1819]
5	ns [t_(17)_ = 0.5871, *p* = 0.5649]
6	ns [t_(17)_ = 0.3392, *p* = 0.7386]
7	ns [t_(17)_ = 0.2614, *p* = 0.7969]
8	ns [t_(17)_ = 0.2461, *p* = 0.8086]
9	ns [t_(17)_ = 0.3445, *p* = 0.7347]
10	ns [t_(17)_ = 0.2594, *p* = 0.7984]
11	ns [t_(17)_ = 0.2367, *p* = 0.8157]

##### FAAH

DNA methylation was analyzed at 9 CpG sites within the promoter region of the FAAH gene was analyzed. A two tailed, unpaired *t*-test of mean methylation levels across the analyzed 9 CpG sites revealed no statistically significant difference between the CON and ELS groups [t_(17)_ = 1.198, *p* = 0.2473; Figure 4B]. Also, for each of the analyzed 11 CpG sites individually no statistically significant differences between the CON and ELS groups were detected (Figure 5B; see Table 2 for details).

**Table 2:** Unpaired *t*-test analysis of 9 individual CpG sites within the promoter region of the FAAH gene.

Individual CpG sites	*t*-test results (non-significant = ns)
1	ns [t_(17)_ = 0.6483, *p* = 0.5254]
2	ns [t_(17)_ = 1.108, *p* = 0.2835]
3	ns [t_(17)_ = 0.4196, *p* = 0.68]
4	ns [t_(17)_ = 0.499, *p* = 0.6242]
5	ns [t_(17)_ = 0.3351, *p* = 0.7416]
6	ns [t_(17)_ = 1.806, *p* = 0.0886]
7	ns [t_(17)_ = 0.8021, *p* = 0.4336]
8	ns [t_(17)_ = 1.057, *p* = 0.3055]
9	ns [t_(17)_ = 1.862, *p* = 0.08]

### Histone modifications

#### ELS exposure induced decreased global histone 3 acetylation

**Figure 6:**
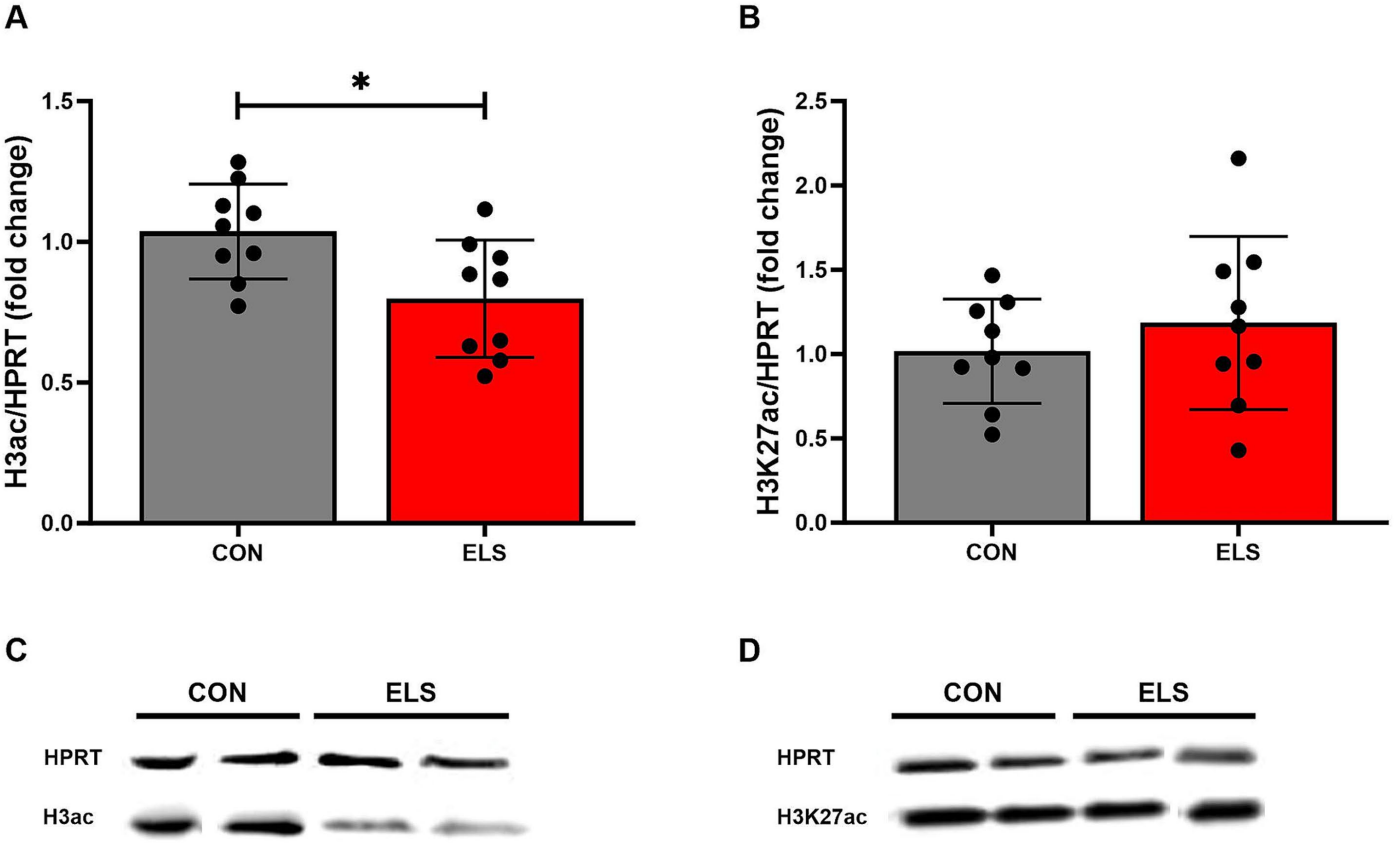
ELS-induced changes on the histone 3 global acetylation in the medial prefrontal cortex (mPFC) of adult male rats. **(A)** H3 acetylation levels, **(B)** H3K27 acetylation levels, **(C,D)** Representative Western Blots from 2 control and two ELS animals, respectively. H3ac/HPRT and H3K27/HPRT ratio is presented as fold change relative to the mean value of the respective control group. Significant results from two-tailed, unpaired *t*-tests are indicated as *p ≤ 0.05. All bars represent mean ± SD. CON, control (*n* = 9); ELS, early life stress (*n* = 9).

For the global histone 3 acetylation levels, two tailed, unpaired *t*-test analysis comparing ELS males to controls showed a significant decrease in the ELS group, when compared to the CON group [t_(16)_ = 2.671, *p* = 0.0167; Figures 6A,C].

#### ELS exposure did not affect H3K27 acetylation

For the histone 3 lysine 27 acetylation levels, an unpaired *t*-test analysis comparing ELS males to controls revealed no significant difference [t_(16)_ = 0.84, *p* = 0.4132; Figures 6B,D].

#### No change in promoter specific H3 acetylation after ELS

Unpaired *t*-test analysis of histone H3 acetylation at the CB1R promoter region revealed no significant difference between ELS males and controls [t_(10)_ = 1.052, *p* = 0.3177; Primer 1], [t_(10)_ = 0.0572, *p* = 0.9555; Primer 2; results not shown; CON (*n* = 6), ELS (*n* = 6)]. Similarly, unpaired *t*-test analysis of histone H3 acetylation at the FAAH promoter region revealed no statistically significant differences between ELS males and controls [t_(10)_ = 1.061, *p* = 0.3137; results not shown].

## Discussion

Childhood adversities have been linked to the emergence of mental disorders in adulthood, including anxiety and depression (Green et al., 2010; Shonkoff and Garner, 2012; Berens et al., 2017). Rodent models of neuropsychiatric disorders emphasize environmental factors, in particular stress in early life, as an underlying cause for maladaptive mechanisms (Wang et al., 2012; Gröger et al., 2016; Bondar et al., 2018; Ishikawa et al., 2015). However, it is also known that ELS, as well as exposure to multiple stressors at later developmental stages may lead to improved stress coping and promote behavioral and cellular adaptations that enhance resilience. These effects depend on the type and duration of stress (Santarelli et al., 2017; Jaric et al., 2019; Maniam et al., 2014; Daskalakis et al., 2013) and the developmental stage of the individual at the time of stress exposure (Lupien et al., 2009; Bock et al., 2005).

In our study behavioral profiling unveiled long-term effects of ELS exposure on the behavioral phenotype of male rats in adulthood. Specifically, adult ELS males displayed impaired social behavior, anhedonia (symptom of depressive-like behavior), and reduced anxiety. Supporting the hypothesis that these ELS-induced behavioral changes may at least in part be mediated by changes in endocannabinoid function, we observed a downregulation of CB1R and FAAH gene expression in the mPFC of ELS exposed adult males. Finally, we addressed the hypothesis that this long-term ELS-induced “re-programming” of gene expression may be orchestrated via epigenetic modifications. While DNA methylation was not affected by ELS exposure, global histone 3 acetylation was decreased in ELS males, which indicates that this mechanism may contribute to the observed decreased gene expression.

### Reduced sociability and increased depressive-like behavior in adult male rats after ELS exposure

Various ELS paradigms have been applied in preclinical studies to study the immediate and long-term impact on the behavioral, and on the molecular and physiological level in the brain. One established paradigm involves rearing animals with reduced bedding material during the second postnatal week, resulting in abnormal maternal care characterised by fragmented and unpredictable maternal behavior as well as heightened sensitivity to stress in offspring and impaired hipocampus-mediated spatial learning and dendritic atrophy in the hippocampal formation (Gilles et al., 1996; Avishai-Eliner et al., 2001; Ivy et al., 2008; Ivy et al., 2010). The limited bedding paradigm offers a controlled environment to study the long-term effects of ELS, providing valuable data with direct implications for human research and treatment approaches. The persistence of social and emotional deficits in ELS-exposed rats throughout their lifespan suggests that early interventions may be crucial in preventing long-term mental health consequences in humans. By understanding how ELS influences behavior and brain development in animal models, researchers can develop targeted interventions to mitigate these effects, potentially reducing the incidence of depression, social anxiety, and related disorders in humans (Molet et al., 2014; Baram et al., 2012; Rice et al., 2008; Joshi and Onaivi, 2021). In the current study we applied a slightly modified maternal neglect paradigm in rats that has previously been shown to lead to impaired sociability, anxiety-like and depression-like behavior in adolescent and adult rats (Alteba et al., 2016, 2020, 2021; Portugalov et al., 2022; Demaili et al., 2023).

Along this line, the present study provides further evidence that ELS-exposed male rats develop anhedonic behaviour lasting into adulthood as measured in the saccharine preference test. Anhedonia, the inability or loss of pleasure in a wide range of activities, is among the core symptoms to diagnose a major depressive disorder in humans according to the DSM-5 (American Psychiatric Association, 2013; Hamilton, 1967). Anhedonia can be divided into subcomponents, consummatory anhedonia, which is the emotional “liking” component reflected by reduced hedonic response and motivational anhedonia, the “wanting” component, reflected by loss of interest or motivation (Treadway and Zald, 2011). Since in animal models these specific diagnostic criteria cannot be applied completely, a number of behavioral tests have been developed to test for specific aspects of depressive-like behavior that reflect symptoms of the mental disorder in humans (Gencturk and Unal, 2024). In our study, anhedonia was reflected by reduced preference for saccharine. The saccharine or sucrose preference test is one of the most common tests for assessing anhedonia and covers the consummatory component, but to some extend also motivational aspects observed in our previous studies, where the results from forced swim test (FST) unveiled symptoms of despair and helplessness in ELS-exposed adult male and female rats (Portugalov et al., 2022; Alteba et al., 2020).

In the present study, we also observed that adult ELS-exposed male rats showed disturbed social behavior, as indicated by results of the social preference test, where ELS-exposed rats spent less time with social partner. This observation replicates findings from our previous studies, where we have found reduced sociability of ELS exposed male and female rats, in adolescence and also in adulthood (Demaili et al., 2023; Alteba et al., 2020, 2021). This dysfunctional social behavior was detectable as early as PND 20, and appears to persist throughout lifespan (Raineki et al., 2012, 2015). Impairments in social behavior or interaction can be a result of the combination of symptoms for depressive disorders according to the DSM-5 (American Psychiatric Association, 2013). In addition, the observed ELS-induced impairment of social behavior might be indicative of symptoms of autistic spectrum disorder, in which the endocannabinoid system has been shown to play a major role (Joshi and Onaivi, 2021).

Interestingly, the observed deficits in socio-emotional behavior were not paralleled by symptoms of enhanced anxiety in the open field test (OF), which is in line with findings from our previous study applying the same ELS-paradigm (Alteba et al., 2020) and a study, which reported that ELS-induced anhedonia was not accompanied by measures of anxiety (Molet et al., 2016). In contrast, the present results of the EPM test revealed reduced anxiety in ELS-exposed males. A similar finding was observed in a study, using maternal separation as ELS paradigm, reporting reduced anxiety in adolescent males exposed to ELS (Wang et al., 2015). With regard to the interpretation of these results it remains to be clarified whether the reduced anxiety symptoms observed in ELS-exposed animals may be related to increased risk-taking behavior and/or may indicate positive adaptations to early adverse events leading to situation-specific resilience.

### ELS exposure downregulated endocannabinoid function in the mPFC of adult male rats

Numerous studies have shown that adverse environmental challenges in early life, in particularly stress, influence the functional development of the endocannabinoid system in different brain areas (Marco et al., 2014; Moussa-Tooks et al., 2020; Hill et al., 2019; Romano-López et al., 2016; Amancio-Belmont et al., 2020). Consistent with these findings, our study provides evidence that ELS-induced behavioral changes are paralleled by altered endocannabinoid function. For both, CB1R and FAAH mRNA, expression levels in the mPFC were reduced in adult ELS-exposed males compared to unstressed control animals. These results are in accordance with previous observations (Alteba et al., 2021), where we reported that adult ELS-exposed male rats showed a decrease of CB1R expression in the mPFC-hippocampal circuit, correlating with the behavioral outcome, including despair-like behavior. Other studies using maternal separation as the ELS paradigm also reported reduced CB1R binding in the mPFC of male rats during adolescence and adulthood (Hill et al., 2019; Goldstein Ferber et al., 2021).

There is considerable evidence that eCB signaling is critically involved in the regulation of stress responses. Reduced activity of the main regulatory elements of the endocannabinoid system is related to dysfunctional stress regulation, fear responses and anxiety symptoms (Hillard, 2014; Jenniches et al., 2016; Patel et al., 2017). Regarding the functional/physiological interpretation of our molecular data, it is important to note that CB1R expression is particularly prominent in brain areas, especially the PFC, that are involved in anxiety and stress regulation, behavioral flexibility and adaptation as well as in learning and memory functions. ECS is a homeostatic system; therefore it is safe to suggest that CB1R and FAAH downregulation in the mPFC leads to dysregulated endocannabinoid signalling in this brain area and a consequentially higher risk for the development of mood disorders and psychiatric diseases. The altered behavior can potentially be rescued with a pharmacological treatment approach, as we have previously shown (Mizrachi Zer-Aviv et al., 2022). In this study, we had male rats exposed to a single foot shock. Rats performed poorly in the behavioral tests battery, including low saccharine consumption and higher immobility time in the FST, as well as lower scores in social preference and recognition tests. Pharmacological intervention with FAAH inhibitor, URB597, has restored these behaviors, specifically social behavior and depressive-like symptoms. Similarly, for ELS males, we have found that URB597 applied in adolescence restored ELS-induced freezing in FST and social recognition (Portugalov et al., 2022). Both of our previous studies imply ECS involvement in depressive-like behavior and social disorders and suggest potential future therapy directions.

CB1R is also involved in synaptic plasticity mechanisms such as long-term potentiation (LTP; Abush and Akirav, 2013) and long-term depression (LTD; Castillo et al., 2012). Presynaptic CB1Rs are retrogradely activated by postsynaptically released 2-AG or AEA and thereby can inhibit the release of GABA (resulting in disinhibition) and/or glutamate (reducing excitatory activity), thus modulating the essential excitatory-inhibitory balance in neuronal networks. As a consequence, disturbed CB1R signaling may hinder the mentioned plasticity processes, leading to difficulties in adapting to new environmental challenges. However, the methodology used in this study does not allow a cellular or synaptic resolution and thus it can only be speculated that the observed ELS-induced downregulation of CB1R as well as FAAH expression in the mPFC might indicate a dysbalance of excitation/inhibition in this brain region, potentially affecting short- and long-term synaptic plasticity (Scheyer et al., 2023). Moreover, it is tempting to speculate that the ELS-induced downregulation of FAAH expression in the mPFC, observed in the ELS-exposed males, may be associated with the measured symptoms of reduced anxiety. This hypothesis is supported by pharmacological studies, which show that suppression of FAAH activity exerts anxiolytic effects (Fidelman et al., 2018; Segev et al., 2014; Griebel et al., 2018; Marco et al., 2015; Haller et al., 2009; Scherma et al., 2008). Since CB1Rs are located not only on presynaptic terminals of neurons but also on astrocytes (Yin et al., 2019; Scheyer et al., 2023), their downregulation may also distrupt astrocytic functions, including the modulation of synaptic transmission and neuroinflammatory responses. On the other hand, FAAH, an enzyme responsible for degrading AEA, and a negative regulator of ECS signalling, is located in postsynaptic structures as well as in astrocytes and microglia. Reduced FAAH expression could impact neuroinflammatory pathways, which are implicated in various psychiatric disorders. Taken together, the concurrent reduction of both CB1R and FAAH suggests a complex dysregulation of the endocannabinoid system, which may impair synaptic plasticity and contribute to maladaptive stress responses and the development of mental disorders.

In this study we focused on the long-term effects of ELS exposure on adult male rats, whereas our previous study focused on female rats (Demaili et al., 2023). Comparing the results for male and female rats behavioral profiling revealed that the percentage of affected/vulnerable subjects was higher in ELS-females (50% affected) than in ELS-males (28% affected). This is consistent with multiple studies, reporting that females have an increased risk for the development of mental disorders following adverse experiences (Whitaker et al., 2021; Rubinow and Schmidt, 2019; Goodwill et al., 2019). Moreover, our two studies revealed that males and females appear to develop slightly different phenotypes regarding anxiety behavior. While males in the present study displayed reduced anxiety levels without motor hyperactivity, female rats in the previous study showed higher motor activity (perhaps indicative of hyperactivity symptoms) but no changes in anxiety levels (Demaili et al., 2023). In contrast, no sex-differences were observed in depressive-like and social behavior, as both males (present study) and females (Demaili et al., 2023) displayed similar symptoms of anhedonia and reduced social interest. Regarding the detected ELS-induced changes in the endocannabinoid system we found opposing sex-specific effects. The adult males in the present study exibited a downregulation of CB1R and FAAH expression in the mPFC, whereas adult ELS females showed an upregulation of CB1R and no changes of FAAH expression in the mPFC (Demaili et al., 2023). This suggests a sexual dimorphism in the developmental trajectories and maturation of the ECS as a potential explanation for the observed sex-specific effects. Postnatally, the ECS develops at different rates in the male and female rat brain. There is a high number of CB1Rs until the pruning process in late adolescence and adulthood, with females having the highest numbers of CB1R at PND30 and males at PND40 (Rodríguez de Fonseca et al., 1993). ECS dimorphism is particularly evident in the PFC, as seen in another rat study (Bernabeu et al., 2020). Endocannabinoid-mediated maturational trajectories were active in juvenile females, but lacking in males, where they emerged later in puberty.

### ELS affects global acetylation levels of histone H3

Increasing evidence reveals that the perinatal adverse experiences influence brain and behavior through both transient and permanent epigenetic mechanisms (Bock, 2024; Szyf, 2021; Bock et al., 2015; Braun et al., 2020; Alyamani and Murgatroyd, 2018; Li et al., 2020; Uchida et al., 2010). Epigenetic modification represents a crucial regulator for the fine-tuning of gene expression and the classical view is that enhanced DNA-methylation (hypermethylation) leads to decreased or even silenced gene expression, whereas increased histone acetylation is associated with open, transcriptionally active chromatin resulting in elevated gene expression (Gräff and Mansuy, 2008; Allis and Jenuwein, 2016; Greenberg and Bourc'his, 2019). We hypothesized that ELS “reprograms” the expression of the CB1R and FAAH genes, and that the changes observed in ELS-exposed adult males are due to epigenetic alterations. While we did not detect significant changes in DNA methylation within the promoter regions of the CB1R and FAAH genes, we observed significantly lower global histone H3 acetylation levels in ELS-exposed males compared to unstressed, control males. This supports our working hypothesis that the observed ELS-induced downregulation of CB1R and FAAH gene expression is epigenetically regulated.

This interpretation is corroborated by other studies that show post-translational histone acetylation regulates ELS-induced changes of gene transcription, replication and DNA structure. For example, in one of our previous studies in mice (Xie et al., 2013), where 3 days of maternal separation was applied as mild ELS exposure, we observed a rapid increase in acetylation levels of H3 and H4 immediately after the last separation period on PND 16. This increased histone acetylation was directly associated with increased expression levels of the synaptic plasticity genes Arc and Egr1 in the hippocampus.

The effect on histone acetylation appears to be transient. In contrast to the immediate increase, applying the same stress paradigm, we found a decrease in H3 acetylation levels in the mPFC of adult ELS-males detected in the present study. Similarly, a study that applied a stronger stressor, chronic unpredictable stress, reported that male mice had lower histone 3 lysine 9 acetylation (H3K9ac) levels associated with CB1R gene expression (Lomazzo et al., 2017).

However, it should be noted that nChIP analysis did not detect significant ELS-induced promoter-specific changes of H3 acetylation within the promoter regions of the CB1R and FAAH genes. We have taken into consideration that only circumscribed sequences within the promoter regions were analyzed and that regulatory elements controlling the interaction between histone H3 acetylation and chromatin might be located in other parts of the promoter or even at other regions such as enhancer (Engelen et al., 2015; Ji et al., 2015; Chen et al., 2019).

## Conclusion

The main conclusion from the discussion of our study is that early life stress (ELS) has significant long-term effects on the behavioral and molecular phenotype of adult male rats. Specifically, ELS-exposed males exhibit impaired social behavior and anhedonia. These behavioral changes are accompanied by a downregulation of CB1R and FAAH gene expression in the medial prefrontal cortex (mPFC), potentially indicating a disbalance in excitatory/inhibitory signaling resulting in impaired synaptic plasticity. Additionally, decreased global histone H3 acetylation levels suggest that epigenetic mechanisms might mediate the observed ELS-induced gene expression changes. However, it should be noted that the correlations observed in our study might in addition also include other downstream molecular changes, which were not analyzed here. Furthermore, it remains an open question which epigenetic mechanisms are specifically mediating the observed changes of the endocannabinoid system, influenced by ELS exposure. Future investigations should answer whether the observed gene expression and histone modifications may contribute to the development of specific behavioral phenotypes, that can be classified as resilient or vulnerable.

## Data Availability

The original contributions presented in the study are included in the article/supplementary material, further inquiries can be directed to the corresponding author.
